# Dietary Approaches to Stop Hypertension score is associated with depressive symptoms among Chinese adults: a cross-sectional study

**DOI:** 10.3389/fnut.2026.1913667

**Published:** 2026-10-21

**Authors:** Ping Li, Yue Li, Yanru Zhao, Xinna Fan, Li Liu, Ru Zhang, Tingting Gong, Jiarong Zhang, Xiumei Li, Cuirong Li, Longye Zhang

**Affiliations:** 1 Department of Nutrition, First Hospital of Qinhuangdao, Qinhuangdao, China; 2 Medical Affairs Office, First Hospital of Qinhuangdao, Qinhuangdao, China; 3 Operating Room, First Hospital of Qinhuangdao, Qinhuangdao, China; 4 Department of Traditional Chinese Medicine, First Hospital of Qinhuangdao, Qinhuangdao, China; 5 Center for Disease Control and Prevention, Qinhuangdao, China; 6 Department of General Practice, First Hospital of Qinhuangdao, Qinhuangdao, China; 7 Department of Nutrition, Qinglong Manchu Autonomous County Hospital, Qinhuangdao, China; 8 Department of Nephrology, First Hospital of Qinhuangdao, Qinhuangdao, China

**Keywords:** Chinese adults, DASH score, depressive symptoms, dose-response relationship, OR

## Abstract

**Background:**

The relationship between diet and depressive symptoms is increasingly recognized. However, studies of the association between the Dietary Approaches to Stop Hypertension (DASH) diet and depressive symptoms are limited, and data from Chinese adults are lacking.

**Objective:**

The goal of this study was to explore the association between the DASH score and depressive symptoms in Chinese adults and to analyze the dose-response relationship and threshold effect.

**Methods:**

A total of 3,465 adults were enrolled in this study. Dietary intake was assessed using a food frequency questionnaire, and then the DASH score was calculated. Depressive symptoms were evaluated using the Zung Self-Rating Depression Scale. Subsequently, multivariable logistic regression analysis was used to assess the association between DASH score and depressive symptoms. Restricted cubic spline and two-piecewise logistic regression models were applied to explore dose-response relationships and threshold effect.

**Results:**

Logistic regression analysis showed a significant negative association between the DASH score and depressive symptoms (*p* for trend = 0.045), which remained stable across different adjusted models. A significant non-linear dose-response relationship was observed (*p* for non-linearity = 0.001). An exploratory inflection point was identified at a DASH score of 4.124 (95% CI: 2.050–6.450) in this cross-sectional sample. Below this value, a higher DASH score was associated with lower odds of depressive symptoms (OR = 0.397, 95% CI: 0.261–0.605, *p* < 0.001); above this value, the association was not significant.

**Conclusion:**

To the best of our knowledge, this is the first study to identify a significant inverse association between the DASH score and depressive symptoms in Chinese adults.

## 1 Introduction

Depression is a major public health issue worldwide; it affects more than 300 million people and is a leading cause of disability-adjusted life years (1, 2). In China, the prevalence of depression among adults has been on the rise, with approximately 53 million people affected (3), but the treatment rate is surprisingly low (4). Depressive symptoms not only impair patients' quality of life but also increase family care burden and socioeconomic costs (1, 3). Therefore, identifying modifiable factors associated with depressive symptoms is of great public health significance.

In recent years, the role of diet and nutrition in relation to depressive symptoms has received widespread attention (5, 6). A meta-analysis that included 41 studies (7) showed that the Mediterranean diet and lower Dietary Inflammatory Index are significantly associated with lower odds of depressive symptoms. However, the Dietary Approaches to Stop Hypertension (DASH) diet—a well established healthy dietary pattern (8)—has received limited attention in depressive symptoms research. To date, only a few studies (9–11) have examined this association, and they have yielded inconsistent results. Perez-Cornago et al. (9) and Valipour et al. (10) reported an inverse association between the DASH diet and depressive symptoms, whereas Meegan et al. (11) found no significant association between the two. Importantly, existing evidence is mainly derived from Western populations, and relevant data from Chinese adults remain scarce.

The DASH diet is characterized by high intake of fruits, vegetables, whole grains, low-fat dairy products, and lean protein, and its effects have been well documented in relation to cardiovascular health (12–15). However, the association between the DASH diet and depressive symptoms, especially its applicability to the Chinese population, dose-response relationship, and threshold effect, remains unclear to date. Thus, the goals of this study were to investigate the association between the DASH score and depressive symptoms among Chinese adults and to explore the dose-response relationship and threshold effect.

## 2 Materials and methods

### 2.1 Participants

Participants were recruited from individuals undergoing annual physical examinations at the First Hospital of Qinhuangdao, China, which is the only comprehensive tertiary Grade A hospital in the region with the largest and most comprehensive physical examination center. This study was approved by the Ethics Committee of the First Hospital of Qinhuangdao and performed in accordance with the Declaration of Helsinki. Written informed consent was obtained from all participants before enrollment.

In total, 4,632 subjects were recruited from January to November 2025, and all of them received a questionnaire on demographics, dietary intake, lifestyle, and health status. After excluding participants with incomplete dietary data (*n* = 537), daily energy intake at the extreme 0.5% at each end of the distribution (*n* = 41), incomplete depressive symptoms scale (*n* = 198), and missing covariates (*n* = 391), 3,465 participants were included in the analysis (Figure 1). No significant differences in the baseline characteristics were detected between included participants (*n* = 3,465) and excluded participants (*n* = 1,167) (Supplementary Table 1).

**Figure 1:**
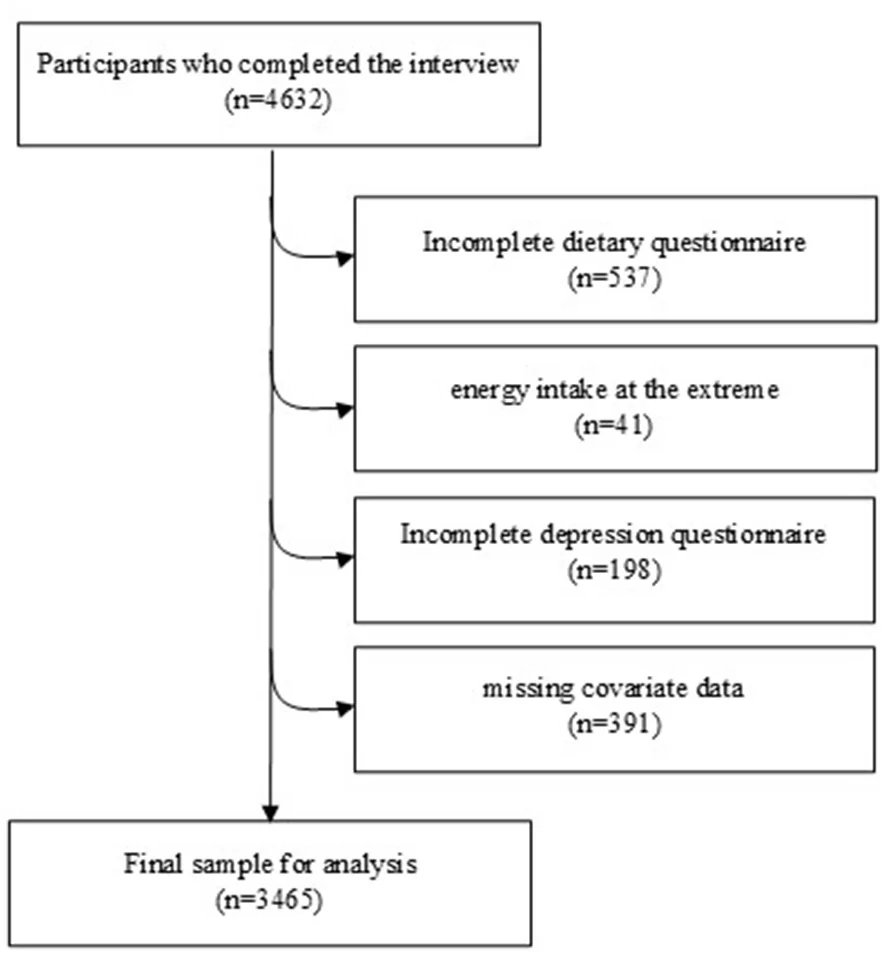
Flow chart of the screening process.

### 2.2 Dietary assessment and DASH score

Dietary intake was assessed using a validated semi-quantitative food frequency questionnaire (FFQ) (16). The FFQ consisted of 81 food items with specified serving sizes for each food. Seven response categories were provided for consumption frequency: (i) rarely or never; (ii) less than once per week; (iii) once per week; (iv) 2–3 times per week; (v) 4–6 times per week; (vi) once per day; and (vii) more than twice per day. Participants recalled their consumption frequency of each food item over the past month. The average daily intakes of foods, nutrients, and total energy were calculated by converting the selected frequency for each item into a daily equivalent, using the China Food Composition Table as the reference database.

The DASH score was computed using the method described by Mellen et al. (17), which is based on nine nutrient targets: total fat, saturated fat, protein, dietary fiber, cholesterol, calcium, magnesium, sodium, and potassium. These targets are derived from the DASH diet and thus provide a valid measure of adherence to this food-based pattern. All nutrient intakes were standardized per 1,000 kcal to eliminate confounding bias induced by inter-individual discrepancies in total energy intake. For each component, participants received 1 point if they met the DASH target, 0.5 points if they achieved the intermediate goal, and 0 points if they met neither. The total DASH score was calculated as the sum of the nine component scores (range: 0–9). Supplementary Table 2 presents the scoring criteria for the nine nutrients (18, 19).

### 2.3 Assessment of depressive symptoms

Depressive symptoms were assessed using the Chinese version of the Zung Self-Rating Depression Scale (SDS) (20), which is a 20-item self-report questionnaire that has been validated in Chinese populations (20, 21). Each item is rated on a 4-point Likert scale ranging from 1 to 4, with total scores ranging from 20 to 80; higher scores indicate greater depressive symptom severity. The Chinese version of the SDS has demonstrated good reliability, with Cronbach's α coefficients ranging from 0.70 to 0.92 across diverse Chinese populations (22, 23). In the present study, the Cronbach's α coefficient was 0.81. Furthermore, Xia et al. (20) demonstrated that an SDS cutoff of 45 achieved a sensitivity of 83.6% and specificity of 96.4% for Diagnostic and Statistical Manual of Mental Disorders in diagnosis of depressive symptoms. In accordance with previous studies (20, 24–27), we used a cutoff of ≥ 45 to identify depressive symptoms.

### 2.4 Other covariates

Anthropometric measures (height and weight) were measured following standardized protocols. Participants were measured wearing light clothing and without shoes. Body mass index (BMI) was calculated as weight in kilograms divided by the square of height in meters (kg/m^2^).

Sociodemographic and lifestyle-related data were also collected and included sex, age, marital status, cohabitants, education, employment status, history of physical illness, smoking, drinking status, and physical activity. Marital status was classified as married or unmarried; cohabitants was categorized as living alone or living with others; educational level was divided into below college and college or above; and employment status was grouped into managers or professionals and others. History of physical illness was assessed using binary “yes/no” responses to relevant questions. Smoking status was categorized as never smoker, former smoker, or current smoker. Drinking status was classified by frequency as never, former, sometimes, or daily drinking.

Physical activity level was assessed using the short form of the International Physical Activity Questionnaire (28). Information on walking, moderate-intensity, and vigorous-intensity activities in the past week was collected. Weekly metabolic equivalent (MET)-hours were calculated as: MET coefficient × activity duration (hours) × frequency (days). Total physical activity level was determined by summing the MET scores across all activity types.

### 2.5 Statistical analysis

Categorical variables are presented as proportions (%), and continuous variables are described as mean ± standard deviation or median (interquartile range), as appropriate. Between-group comparisons were conducted using one-way analysis of variance, the Kruskal-Wallis test, or the chi-square test, depending on the distributional characteristics of the variables.

Multivariate logistic regression models were employed to assess the association between the DASH score and depressive symptoms, and odds ratios (ORs) and 95% confidence intervals (CIs) were calculated. Model 1 was adjusted for age. Model 2 was further adjusted for sex, BMI, marital status, and education. Model 3 was fully adjusted, including age, sex, BMI, marital status, education, cohabitants, employment status, history of physical illness, smoking and drinking status, energy intake, and physical activity.

Restricted cubic spline regression was performed to explore the dose-response relationship between the DASH score and depressive symptoms after adjusting variables in Model 3, with four knots placed at the 5^th^, 35^th^, 65^th^, and 95^th^ percentiles of the DASH score distribution and the median as the reference. Furthermore, a two-piecewise logistic regression model with smoothing was used to analyze the association threshold between the DASH score and depressive symptoms. Likelihood-ratio tests and 1,000 bootstrap resampling replications were employed to identify the inflection point.

In addition, subgroup analyses were performed, with data stratified by sex, age (< 45 years vs. ≥45 years), BMI (< 24 kg/m^2^ vs. ≥ 24 kg/m^2^), and educational level (below college vs. college or above). Heterogeneity among subgroups was assessed by multivariate logistic regression, and interactions between subgroups and the DASH score were examined by likelihood ratio testing.

All analyses were performed using R Statistical Software (Version 4.2.2, http://www.R-project.org, The R Foundation) and the Free Statistics analysis platform (Version 2.2, Beijing, China) (29). All *p* values were two-tailed, and *p* < 0.05 was considered to be significantly different.

## 3 Results

### 3.1 Participant characteristics

Table 1 summarizes the baseline characteristics of all participants. Of the 3,465 participants included in the final analysis, 460 (13.3%) presented with depressive symptoms. Compared to participants with depressive symptoms, those without depressive symptoms exhibited significantly higher DASH scores (*p* = 0.029) and higher physical activity levels (*p* = 0.001), as well as lower proportions of living alone (*p* = 0.009) and history of physical illness (*p* = 0.031). Participants without depressive symptoms were also more likely to be managers or professionals (*p* = 0.024). No other between-group differences reached statistical significance.

**Table 1:** Participant characteristics by depressive symptoms.

Characteristics	Without depressive symptoms (*n* = 3,005)	With depressive symptoms (*n* = 460)	Standardized differences	*p*-Value^*^
Sex (%)			0.06 (−0.04, 0.15)	0.256
Male	1042 (34.68)	172 (37.39)		
Female	1963 (65.32)	288 (62.61)		
Age (years)	41.93 ± 11.28	42.27 ± 10.92	0.03 (−0.07, 0.13)	0.537
Body mass index (kg/m^2^)	24.09 ± 3.78	24.11 ± 3.71	0.01 (−0.09, 0.10)	0.900
Physical activity (mets × h/week)	16.50 (8.31, 31.90)	13.53 (7.70, 24.55)	0.13 (0.02, 0.25)	0.001
Energy intake (kcal/day)	2136.38 ± 940.56	2158.88 ± 1249.02	0.02 (−0.08, 0.12)	0.649
Marital status (%)			0.06 (−0.04, 0.16)	0.273
Married	2837 (94.41)	440 (95.65)		
Unmarried	168 (5.59)	20 (4.35)		
Education (%)			0.03 (−0.07, 0.13)	0.511
College or above	2347 (78.10)	353 (76.74)		
below college	658 (21.90)	107 (23.26)		
Employment status (%)			0.14 (0.04, 0.24)	0.024
Managers	219 (7.29)	24 (5.22)		
Professionals	799 (26.59)	103 (22.39)		
Other	1987 (66.12)	333 (72.39)		
Living alone (%)			0.12 (0.03, 0.22)	0.009
Yes	259 (8.62)	57 (12.39)		
No	2746 (91.38)	403 (87.61)		
Physical illness (%)			0.11 (0.01, 0.20)	0.031
Yes	946 (31.48)	168 (36.52)		
No	2059 (68.52)	292 (63.48)		
Smoking status (%)			0.08 (−0.02, 0.18)	0.266
Smoker	462 (15.37)	76 (16.52)		
Ex-smoker	137 (4.56)	28 (6.09)		
Never	2406 (80.07)	356 (77.39)		
Drinker (%)			0.14 (0.04, 0.24)	0.052
Everyday	111 (3.69)	20 (4.35)		
Sometimes	1662 (55.31)	272 (59.13)		
Ex-drinker	216 (7.19)	41 (8.91)		
Never	1016 (33.81)	127 (27.61)		
DASH score	4.38 ± 0.83	4.29 ± 0.87	0.11 (0.01, 0.21)	0.029

Data presented are mean ± SD, median (Q1, Q3), or *N* (%). ^*^Group differences were tested with one-way analysis of variance or Kruskal-Wallis tests for continuous variables and chi-square tests for categorical variables.

### 3.2 Association between DASH score and depressive symptoms

Table 2 presents the association between the DASH score and depressive symptoms. When the DASH score was treated as a continuous variable in multivariable logistic regression models, it was significantly inversely associated with depressive symptoms in the unadjusted model (OR = 0.87, 95% CI: 0.77–0.99). This inverse association persisted after adjustment for age (OR = 0.87, 95% CI: 0.77–0.98) and remained statistically significant with further adjustment for sex, BMI, educational level, and marital status (*p* = 0.025). In Model 3, with additional adjustment for cohabitants, employment status, history of physical illness, physical activity, smoking and drinking status, and energy intake, the inverse relationship remained stable (*p* = 0.034). When the DASH score was categorized into quintiles, the inverse association between the DASH score and depressive symptoms remained consistent across different adjusted models.

**Table 2:** Association between the DASH score and depressive symptoms.

	Crude model		Model 1		Model 2		Model 3	
Variable	OR (95%CI)	*p* value	OR (95%CI)	*p* value	OR (95%CI)	*p* value	OR (95%CI)	*p* value
Continuous								
DASH scores	0.87 (0.77–0.99)	0.029	0.87 (0.77–0.98)	0.023	0.87 (0.76–0.98)	0.025	0.84 (0.72–0.99)	0.034
Categorical								
Quintile 1 ( ≤ 3, *n* = 166)	1.00 (reference)		1.00 (reference)		1.00 (reference)		1.00 (reference)	
Quintile 2 (>3, < 4, *n* = 604)	0.61 (0.4–0.94)	0.024	0.61 (0.4–0.94)	0.024	0.61 (0.4–0.94)	0.024	0.58 (0.34–0.98)	0.043
Quintile 3 (≥4, < 4.5, *n* = 978)	0.41 (0.27–0.62)	< 0.001	0.41 (0.27–0.62)	< 0.001	0.41 (0.27–0.62)	< 0.001	0.39 (0.23–0.65)	< 0.001
Quintile 4 (≥4.5, < 5, *n* = 782)	0.61 (0.4–0.92)	0.019	0.6 (0.4–0.91)	0.017	0.61 (0.4–0.92)	0.02	0.46 (0.27–0.78)	0.004
Quintile 5 (≥5, *n* = 935)	0.48 (0.32–0.73)	0.001	0.48 (0.32–0.73)	0.001	0.48 (0.31–0.73)	0.001	0.46 (0.27–0.77)	0.003
*p* trend		0.045		0.037		0.048		0.044

Crude model, no covariate was adjusted; Model 1, adjusted for age; Model 2, adjusted for age, sex, BMI, education level and marital status; Model 3, adjusted for age, sex, BMI, education level, marital status, cohabitants, employment status, history of physical illness, smoking and drinking status, energy intake, and physical activity.

### 3.3 Threshold effect analysis

Restricted cubic spline regression was used to explore the dose-response relationship between the DASH score and depressive symptoms, and it revealed a significant non-linear association (*p* for non-linearity = 0.001) (Figure 2). With an increase in the DASH score, the odds of depressive symptoms exhibited an overall decreasing trend. Threshold effect analysis identified an exploratory breakpoint at a DASH score of 4.124 (95% CI: 2.050–6.450) in this single-center, cross-sectional sample. In participants with a DASH score below this value, a higher DASH score was associated with lower odds of prevalent depressive symptoms (OR = 0.397, 95% CI: 0.261–0.605, *p* < 0.001). No statistically significant association was observed between the two variables in participants with a DASH score ≥ 4.124 (Table 3). Notably, sample sizes were small at both extremes of the DASH score distribution (Figure 2), which led to correspondingly wide confidence intervals. The trends shown by the curve in these regions need to be further validated by future studies with larger sample sizes.

**Figure 2:**
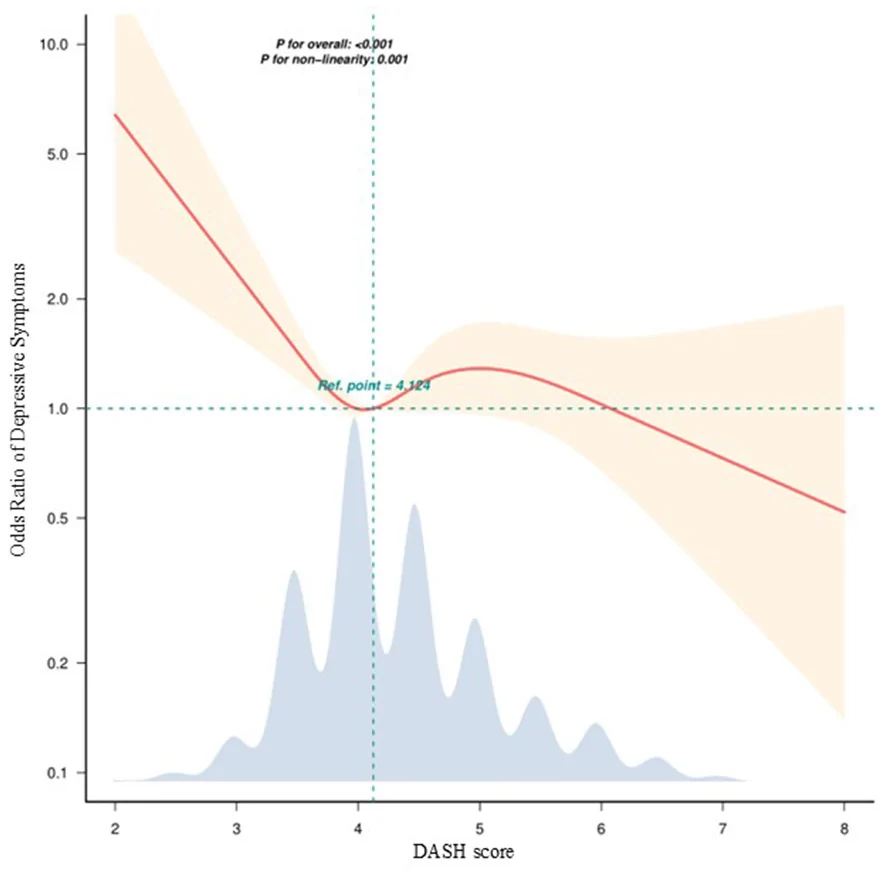
Association between DASH score and depressive symptoms. Adjusted for age, sex, BMI, education level, marital status, cohabitants, employment status, history of physical illness, smoking and drinking status, energy intake, and physical activity.

**Table 3:** Threshold effect analysis of the relationship of the DASH score with depressive symptoms.

DASH score	Adjusted Model	
	OR (95%CI)	*p* value
< 4.124	0.397 (0.261–0.605)	< 0.001
≥4.124	0.953 (0.705–1.288)	0.755
Likelihood Ratio test		0.003

Adjusted for age, sex, BMI, education level, marital status, cohabitants, employment status, history of physical illness, smoking and drinking status, energy intake, and physical activity.

### 3.4 Subgroup analyses

The results of subgroup analyses are presented in Figure 3. Significant inverse associations between the DASH score and depressive symptoms were observed in male participants (OR = 0.78, 95% CI: 0.62–0.99), individuals aged ≥ 45 years (OR = 0.78, 95% CI: 0.64–0.96), those with a BMI ≥ 24 kg/m^2^ (OR = 0.83, 95% CI: 0.70–1.00), and participants with a college degree or higher (OR = 0.86, 95% CI: 0.74–0.99). No significant interaction effects were identified across all subgroups (all *p* for interaction > 0.05). However, several subgroups had confidence intervals crossing the null, and these subgroup findings are exploratory. The apparent differences in significance should not be compared directly, given varying sample sizes and statistical power across strata.

**Figure 3:**
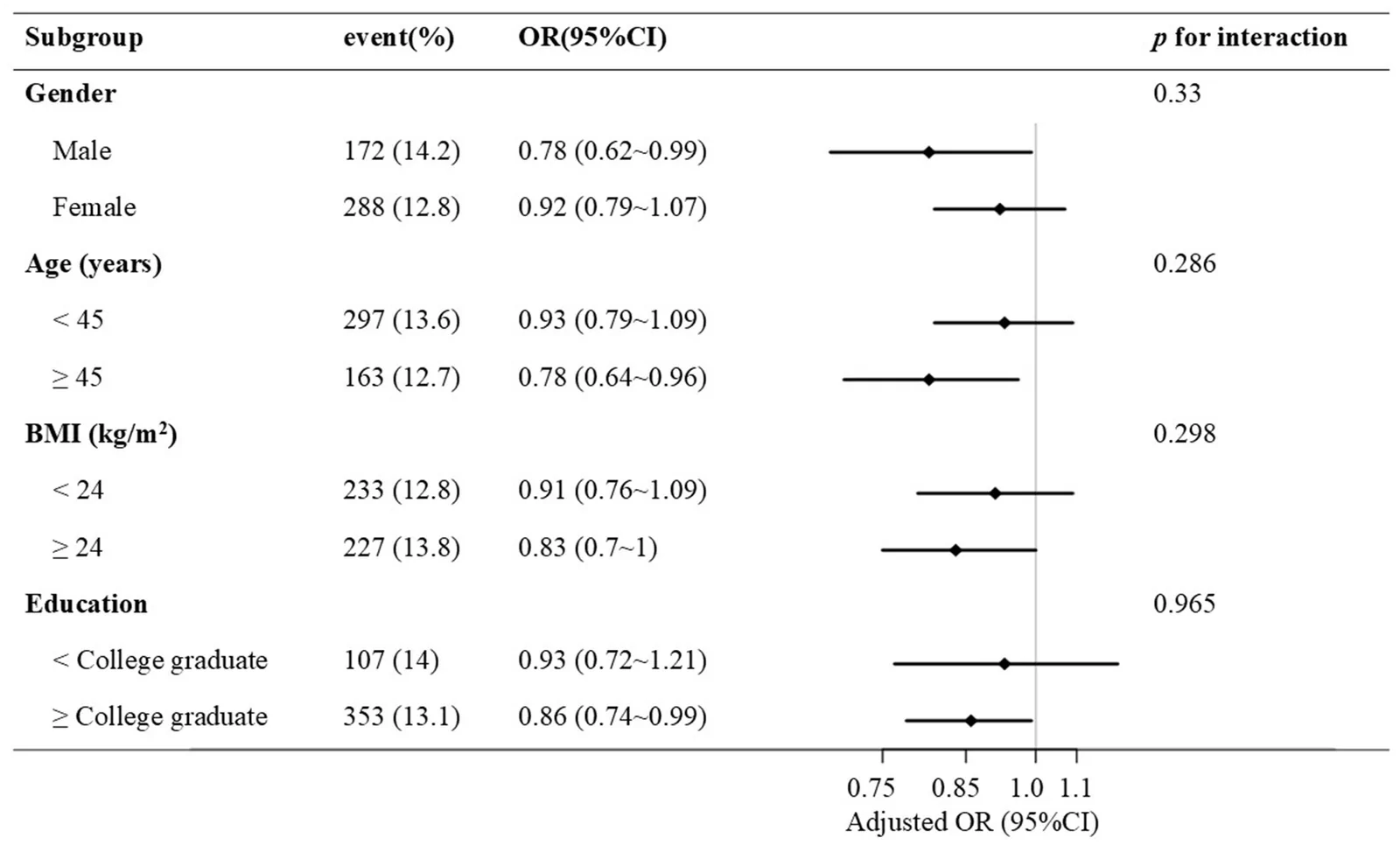
Subgroup analyses of the association between DASH score and depressive symptoms. Adjusted for age, sex, BMI, education level, marital status, cohabitants, employment status, history of physical illness, smoking and drinking status, energy intake, and physical activity (except for the stratification factor itself).

## 4 Discussion

To the best of our knowledge, this is the first study to investigate the association between the DASH diet and depressive symptoms among Chinese adults. Our results revealed a significant inverse association between the DASH score and depressive symptoms, with a non-linear dose-response relationship. An exploratory, population- and sample-specific inflection point was identified at a DASH score of 4.124 (95% CI: 2.050–6.450) in this single-center, cross-sectional sample. Below this value, a higher DASH score was associated with lower odds of prevalent depressive symptoms; above this value, the association was no longer statistically significant.

Our findings are consistent with those of several previous studies. For instance, a Spanish study reported that higher adherence to the DASH diet was associated with a lower risk of depressive symptoms (9), and a similar association was observed in an Iranian population (10). However, our results differ from a study conducted by Meegan et al. (11), in which no significant association was detected in an Irish population. Discrepancies may be attributed to differences in genetic background, baseline dietary patterns, lifestyle, and social environment across populations, as well as variations in depressive symptom assessment tools and cut-off values.

The DASH diet has been well documented to correlate with favorable cardiovascular health outcomes (12–14, 30). The present study extends the scope of this association by examining the association between the DASH diet and mental health, with potential mechanisms involving multiple interconnected pathways. The DASH diet is characterized by high intake of fruits, vegetables, whole grains, and low-fat dairy products. A meta-analysis demonstrated that adherence to a dietary pattern rich in vegetables, fruits, whole grains, and fish, along with reduced animal food intake, was associated with a lower risk of depression (31). Conversely, higher consumption of added sugars and ultra-processed foods was linked to an increased risk of depression (32, 33).

The DASH diet is considered to possess potent antioxidant and anti-inflammatory properties (34). Its abundant dietary fiber, unsaturated fatty acids, and antioxidant-rich foods may be associated with lower levels of systemic inflammation (35) and lower depressive symptom burden through modulation of neurotransmitter metabolism and hypothalamic-pituitary-adrenal axis function (36). In addition, antioxidants from fruits and vegetables may exert potential effects by scavenging reactive oxygen species and reducing oxidative stress-induced neural damage, thereby supporting brain health (37). Consistently, several studies have reported that higher antioxidant levels are associated with lower odds of depressive symptoms (20, 38, 39). From a metabolic perspective, the DASH diet—originally developed for blood pressure control—has been shown to improve multiple metabolic parameters, alleviate systemic inflammatory burden and oxidative stress, and thereby indirectly improve mood and reduce depression risk (40, 41). Furthermore, the gut-brain axis serves as a core bidirectional pathway through which diet may regulate mental health (42). The DASH diet may enhance gut microbial diversity and promote short-chain fatty acid production, which could improve intestinal barrier function, suppress systemic and neuro inflammation, and modulate neurotransmitter synthesis and hypothalamic-pituitary-adrenal axis activity, thereby mitigating depressive symptoms (43, 44).

Psychosocial and behavioral factors also may partly explain the association between the DASH diet and depressive symptoms. Individuals who adhere to healthy dietary patterns tend to possess stronger self-regulation abilities, higher health literacy, and more active lifestyles, and these factors may be associated with lower odds of depressive symptoms (45). Conversely, individuals with depressive symptoms often exhibit irregular eating patterns and increased consumption of energy-dense comfort foods, which may collectively compromise overall dietary quality (46, 47). Given the cross-sectional design of the present study, reverse causation cannot be excluded; that is, depressive symptoms themselves may adversely influence dietary behaviors.

Furthermore, although we adjusted for multiple sociodemographic and lifestyle variables, several unmeasured factors may have introduced residual confounding, including socioeconomic status, psychiatric history, antidepressant use, sleep characteristics, and detailed comorbidity. Although educational attainment was included as a partial proxy for socioeconomic status in our models, it cannot fully capture other socioeconomic dimensions such as household income and wealth, which may jointly influence both dietary quality and mental health. In addition, the absence of data on psychiatric history and antidepressant use precluded adjustment for medication-treated depressive symptoms, which could affect appetite, dietary behaviors, and depressive symptom assessment. Sleep disturbances, which are known to be associated with both poor dietary patterns and mood disorders, were also not assessed (48). Although the inverse association remained stable across Model 1 through Model 3, future prospective studies incorporating richer psychosocial and comorbidity data are warranted to further isolate the independent effect of the DASH dietary pattern on depressive symptoms.

We performed exploratory subgroup analyses. Although some subgroups showed statistically significant associations and others did not, no significant interactions were detected (all *p* for interaction > 0.05), indicating insufficient evidence that the association differs by sex, age, BMI, or education. The inconsistent significance across subgroups may reflect differences in sample size and statistical power rather than true effect modification. Therefore, these findings should not be overinterpreted as indicating that the association is stronger or weaker in any particular subgroup, and they require confirmation in larger, adequately powered studies.

Several strengths of this study should be noted. First, to the best of our knowledge, it is the first to investigate the association between the DASH score and depressive symptoms in Chinese adults, filling the population-specific evidence gap in this field. Second, we clarified the dose-response relationship and threshold effect between the DASH score and depressive symptoms. Third, comprehensive adjustments were made for a wide range of potential confounders, including sociodemographic characteristics, lifestyle factors, and physical activity levels, enhancing the reliability of the effect estimates. Nevertheless, several limitations should be acknowledged. First, the single-center health-examination sample may limit generalizability. Second, the use of self-administered FFQ may introduce measurement error, and the 1-month recall period may not fully capture habitual dietary intake. Third, although we adjusted for multiple covariates, residual confounding from unmeasured factors—such as socioeconomic status, psychiatric history, antidepressant use, and sleep characteristics—cannot be excluded. Fourth, the SDS is a screening tool rather than a psychiatric diagnostic interview. Fifth, the cross-sectional design precludes causal inference. Sixth, the data-derived threshold is exploratory and has not been externally validated. Importantly, this value should not be interpreted as a minimum effective dose, intervention threshold, or clinical target. It is an exploratory, population- and sample-specific inflection point derived from this single-center, cross-sectional sample and requires validation in independent prospective cohorts. Finally, the exclusion of participants with missing data may have introduced selection bias, although the differences between included and excluded participants did not reach statistical significance.

## 5 Conclusions

To the best of our knowledge, this is the first study to report a significant inverse association between the DASH score and depressive symptoms in Chinese adults. Higher DASH scores were associated with lower odds of depressive symptoms in this cross-sectional sample. Future longitudinal studies are warranted to confirm this association.

## Data Availability

The raw data supporting the conclusions of this article will be made available by the authors, without undue reservation.
