# Supplementary material for: Dietary Approaches to Stop Hypertension score is associated with depressive symptoms among Chinese adults: a cross-sectional study
Source: Front Nutr. 2026 Oct 21;13:1913667. doi: 10.3389/fnut.2026.1913667 (PMC13730972; doi:10.3389/fnut.2026.1913667)
Supplement: Supplementary file 1 [file Table_1.docx]

| **Supplementary table 1.** Comparison of baseline characteristics between included and excluded participants | | | | |
| --- | --- | --- | --- | --- |
| Characteristics | Included (n=3,465) | Excluded (n=1,167) | Standardized Differences | *p*-Value* |
| Sex (%) |  |  | 0.029 (-0.037, 0.095) | 0.393 |
| Male | 1214 (35.04%) | 425 (36.42%) |  |  |
| Female | 2251 (64.96%) | 742 (63.58%) |  |  |
| Age (years) | 41.97 ± 11.24 | 41.98 ± 10.52 | 0.001 (-0.066, 0.067) | 0.986 |
| Body mass index (kg/m^2^) | 24.09 ± 3.77 | 24.01 ± 3.72 | 0.022 (-0.044, 0.089) | 0.508 |
| Physical activity (mets × h/week) | 16.50 (8.25, 31.41) | 15.33 (7.70, 31.81) | 0.004 (-0.103, 0.110) | 0.532 |
| Marital status (%) |  |  | 0.037 (-0.040, 0.114) | 0.354 |
| Married | 3277 (94.57%) | 765 (95.39%) |  |  |
| Unmarried | 188 (5.43%) | 37 (4.61%) |  |  |
| Education (%) |  |  | 0.065 (-0.002, 0.131) | 0.059 |
| College or above | 2700 (77.92%) | 940 (80.55%) |  |  |
| below college | 765 (22.08%) | 227 (19.45%) |  |  |
| Employment status (%) |  |  | 0.051 (-0.016, 0.117) | 0.329 |
| Managers | 243 (7.01%) | 91 (7.80%) |  |  |
| Professionals | 902 (26.03%) | 281 (24.08%) |  |  |
| Other | 2320 (66.96%) | 795 (68.12%) |  |  |
| Living alone (%) |  |  | 0.079 (0.002, 0.157) | 0.052 |
| Yes | 316 (9.12%) | 55 (6.96%) |  |  |
| No | 3149 (90.88%) | 735 (93.04%) |  |  |
| physical illness (%) |  |  | 0.049 (-0.018, 0.115) | 0.148 |
| Yes | 1114 (32.15%) | 402 (34.45%) |  |  |
| No | 2351 (67.85%) | 765 (65.55%) |  |  |
| Smoking status (%) |  |  | 0.056 (-0.010, 0.122) | 0.246 |
| Smoker | 538 (15.53%) | 205 (17.57%) |  |  |
| Ex-smoker | 165 (4.76%) | 57 (4.88%) |  |  |
| Never | 2762 (79.71%) | 905 (77.55%) |  |  |
| Drinker (%) |  |  | 0.074 (0.008, 0.140) | 0.184 |
| Everyday | 131 (3.78%) | 47 (4.03%) |  |  |
| Sometimes | 1934 (55.82%) | 614 (52.61%) |  |  |
| Ex-drinker | 257 (7.42%) | 82 (7.03%) |  |  |
| Never | 1143 (32.99%) | 424 (36.33%) |  |  |
| Data presented are mean ± SD, median (Q1, Q3), or N (%). Due to the presence of a small number of missing values for certain variables in the original data of the excluded participants, the effective sample sizes for individual characteristics may vary slightly and do not all sum to 1,167.  *Group differences were tested with one-way analysis of variance or Kruskal-Wallis tests for continuous variables and chi-square tests for categorical variables. | | | | |
|  | | | | |

| **Supplementary table 2**. Nutrients targets for DASH score | | | | | |
| --- | --- | --- | --- | --- | --- |
| Nutrients | DASH target level | Score | DASH intermediate target level | Score | Not meet DASH target level |
| Saturated fat (g) | ≤6% of energy | 1 | >6-11% of energy | 0.5 | >11% of energy |
| Total fat (g) | ≤27% of energy | 1 | >27-32% of energy | 0.5 | >32% of energy |
| Total protein (g) | ≥ 18% of energy | 1 | <18-16.5% of energy | 0.5 | <16.5 of energy |
| Cholesterol (mg/1000 kcal) | ≤71.4/1000 | 1 | >71.4/1000-107.1/1000 | 0.5 | >107/1000 |
| Fiber (g/1000 kcal) | ≥14.8/1000 | 1 | <14.8/1000 – 9.5/1000 | 0.5 | <9.5/1000 |
| Magnesium (mg/1000 kcal) | ≥238/1000 | 1 | <238/1000-158/1000 | 0.5 | <158/1000 |
| Calcium (mg/1000kcal) | ≥590/1000 | 1 | <590/1000-402/1000 | 0.5 | <402/1000 |
| Potassium (mg/1000kcal) | ≥2238 /1000 | 1 | <2238/1000-1534/1000 | 0.5 | <1534/1000 |
| Sodium (mg/1000kcal) | ≤1143/1000 | 1 | >1143/1000-1286/1000 | 0.5 | >1286/1000 |
